# Digital Tools to Enhance Caregiver-Centered Communication

**DOI:** 10.1093/geroni/igaa057.1893

**Published:** 2020-12-16

**Authors:** Debra Parker Oliver

**Affiliations:** University of Missouri, Columbia, Missouri, United States

## Abstract

While it is recognized that caregiver engagement can improve processes and outcomes of care in gerontology, there are barriers to caregiver centered communication, including limited resources for health systems to devote services specifically to families, geographic distance and lack of time. Digital tools such as social media platforms and video-conferencing introduce opportunities for remote and often asynchronous communication. In this presentation, we discuss findings from two randomized clinical trials that explored digital tools to empower family caregivers. In the first we examined ways to use video-conferencing to enable family caregivers to become virtual team members during hospice interdisciplinary teams, and in the second trial we examine the use of secret Facebook groups to meet informational and emotional needs of family caregivers during episodes of care that are often linked to increased social isolation and loneliness. We discuss challenges and opportunities in designing digital tools to facilitate caregiver engagement and empowerment.

